# Molecular and Mechanical Mechanisms Regulating Ductus Arteriosus Closure in Preterm Infants

**DOI:** 10.3389/fped.2020.00516

**Published:** 2020-08-25

**Authors:** Fahri Ovalı

**Affiliations:** Division of Neonatology, Department of Pediatrics, Istanbul Medeniyet University, Istanbul, Turkey

**Keywords:** ductus arteriosus, oxygen, vasa vasorum, hemodynamics, indomethacin, ibuprofen, acetaminophen, prostaglandins

## Abstract

Failure of ductus arteriosus closure after preterm birth is associated with significant morbidities. Ductal closure requires and is regulated by a complex interplay of molecular and mechanical mechanisms with underlying genetic factors. *In utero* patency of the ductus is maintained by low oxygen tension, high levels of prostaglandins, nitric oxide and carbon monoxide. After birth, ductal closure occurs first by functional closure, followed by anatomical remodeling. High oxygen tension and decreased prostaglandin levels mediated by numerous factors including potassium channels, endothelin-1, isoprostanes lead to the contraction of the ductus. Bradykinin and corticosteroids also induce ductal constriction by attenuating the sensitivity of the ductus to PGE2. Smooth muscle cells of the ductus can sense oxygen through a mitochondrial network by the role of Rho-kinase pathway which ends up with increased intracellular calcium levels and contraction of myosin light chains. Anatomical closure of the ductus is also complex with various mechanisms such as migration and proliferation of smooth muscle cells, extracellular matrix production, endothelial cell proliferation which mediate cushion formation with the interaction of blood cells. Regulation of vessel walls is affected by retinoic acid, TGF-β1, notch signaling, hyaluronan, fibronectin, chondroitin sulfate, elastin, and vascular endothelial cell growth factor (VEGF). Formation of the platelet plug facilitates luminal remodeling by the obstruction of the constricted ductal lumen. Vasa vasorum are more pronounced in the term ductus but are less active in the preterm ductus. More than 100 genes are effective in the prostaglandin pathway or in vascular smooth muscle development and structure may affect the patency of ductus. Hemodynamic changes after birth including fluid load and flow characteristics as well as shear forces within the ductus also stimulate closure. Current pharmacological treatment for the closure of a patent ductus is based on the blockage of the prostaglandin pathway mainly through COX or POX inhibition, albeit with some limitations and side effects. Further research for new agents aiming ductal closure should focus on a clear understanding of vascular biology of the ductus.

Fetal circulation is a unique event, functionally different from pediatric and adult circulation. Ductus arteriosus (DA) is a small, simple vessel with just a few milimeters length and width, but has a profound impact on the survival of the fetus and newborn and plays an essential role in normal postnatal adaptation after birth. It functions as a bridge between two large vessels in fetal life, but sometimes it becomes the “bridge between life and death” in preterm infants. It has baffled scientists since ancient times, and beginning from Galen and Ibn-al Nafis, Vesalius, Leonardo Botallo and William Harvey have commented on its structure and function (1).

During fetal development, cardiovascular septation occurs during days 22–28 of gestation and looping of ventricles completes. At this stage, bilateral aortic arches and bilateral DA form but over the next week, the right aortic arch and ductus involutes. Ductus arteriosus arises from the left sixth embryonic arch; leaves the pulmonary artery very close to the bifurcation point and inserts at the transition zone between the aortic arch and descending aorta, distal to the origin of the left subclavian artery. Usually it has a tubular shape but may be funnel-shaped with a narrow end on the pulmonary side and a wide end on the aortic side (2). Although they all derive from the same embryonic tissue, histological structure of the DA is completely different from that of the aorta or pulmonary arteries. The walls of both great vessels are composed of mainly elastic layers, whereas DA wall is mainly muscular. The inner layer of its wall is composed of longitudinal arranged smooth muscle cells whereas the arrangement of outer layer is mainly circular. On the luminal side, a layer of intimal endothelial cells resides on an internal elastic lamina. This unique structure of the DA sets the stage for the constriction of the vessel after birth.

Ductus arteriosus needs to be remain open in fetal life, in order to sustain the “serial” circulation of the blood. Magnetic resonance studies show that 41% of combined ventricular output passes through the DA in fetal life (3). However, after birth, as the lungs begin to function, a “parallel” circulation is maintained, rendering DA non-functional. Initially it was thought that the ductus was a flabby structure that remained passively open. However, in fetal life, ductus in actively kept dilated by continuous intramural production of prostaglandins (4). The transition from intrauterine to extrauterine life is a critical period and dysregulation of this process may lead to cardiopulmonary instability. Cardiopulmonary adaptation is completed by the closure of DA, followed by closure of ductus venosus and lastly by foramen ovale. In 88% of normal term infants, the ductus is closed by the 8th week of life (5). Preterm infants are born before complete maturation of the cardiovascular system, and they manifest most of the characteristics of fetal life. In a retrospective study of 280 preterm infants who did not receive any therapy directed at closing the ductus, the median time to closure was 71 days in infants born <26 weeks; 13 days in infants born 26–27 weeks, 8 days in infants born 28–29 weeks and 6 days in infants born >30 weeks (6). This implies that extremely preterm infants will be subjected to a period of left-to-right shunting and complications relevant to this shunting.

Ductal closure occurs in two steps: The functional step (vasoconstriction) and the anatomical step (remodeling). Interestingly, the closure of DA resembles the process after vascular injury or atherosclerosis in the adult arteries (7). Understanding different mechanisms which are involved in maintaining ductal tone is essential to comprehend why preterm infants have a high incidence of ductal patency, why some of them do not respond to treatment and where to direct novel therapeutic approaches. On the other hand, it must be stated that most of the experiments and studies toward understanding the physiology of ductal closure are done in animals such as rats or lambs and extrapolated to humans. However, some mechanisms as well as their magnitudes may not be the same in humans.

## Ductal Patency *in utero*

**Figure 1:**
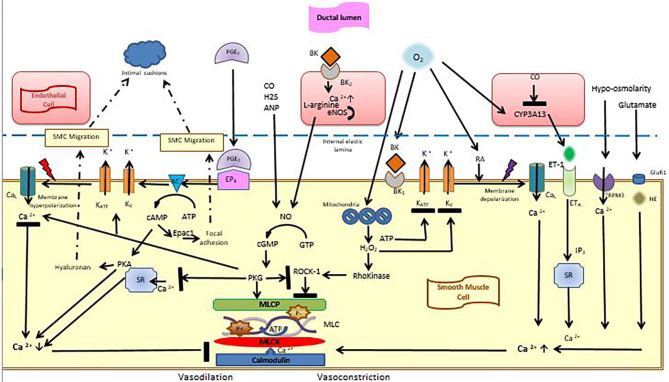
Vasoconstrictive and vasodilatory effects in the ductal smooth muscle cell. Smooth muscle cells are lined with an internal elastic lamina, on which endothelial cells reşide. Vasodilation is maintained by the interaction of PGE2 with its receptor EP4, which triggers potassium outflow through voltage-sensitive potassium channels, which leads to membrane hyperpolarization. Thereby, calcium entry into the cells decreases and binding of calmodulin to MLCK is inhibited. cAMP and cGMP are also effective during this process. After birth, high oxygen tension, which is sensed through the mitochondria triggers the formation of H_2_O_2_ which blocks Rho-kinase pathway as well as potassium channels leading to membrane depolarization and calcium entry into cells. Calcium entry is also stimulated by endothelin-1, hypo-osmolarity and glutamate. Calcium activates MLCK, which leads to MLC phosphorylation and vasoconstriction. cAMP is also effective in smooth muscle migration and intimal cushion formation. AC, adenylyl cyclase; ANP, Atrial Natriuretic peptide; ATP, Adenosine Tri Phosphate; BK, Bradykinin; BK1, Bradykinin receptor; CaL, calcium channels; cAMP, cyclic adenosine monophosphate; cGMP, cyclic guanosin monophosphate; CO, Carbon monoxide; CYP3A13, cytochrome P450; eNOS, endogenous nitric oxide synthase; EP4, Prostaglandin receptor 4; ET-1, Endothelin-1; ETA, endothelin receptor A; epac, exchange protein activated cAMP; H2, Hydrogen sülfite; GluR1, glutamate receptor-1; KATP, KV, Voltage-dependent potassium channels; NE, Norepinephrine; MLC, Myosin Light Chain; MLCK, Myosin Light Chain Kinase; MLCP, Myosin Light Chain Phosphatase; NO, nitric oxide; PKA, cAMP-dependent protein kinase; PGE2, Prostaglandin E2; PKG, cGMP-dependent protein kinase; RA, Retinoic acid; ROCK-1, Rho-associated protein kinase-1; SR, Sarcoplasmic Reticulum; SMC, Smooth Muscle Cell; TRMP3, transient receptor potential melastatin 3 (Figure courtesy of Fahri Ovali).

Smooth muscle tone is regulated by phosphorylation/dephosphorylation of myosin light chain (MLC). MLC is phosphorylated by Calcium/calmodulin-dependent MLC-kinase (MLCK) and dephosphorylated by calcium independent MLC-phosphate (MLCP). Increased cytosolic calcium activates MLCK, which leads to MLC phosphorylation and vasoconstriction (8) (Figure 1). Patency of DA is regulated by counteractive forces causing vasodilation or vasoconstriction. In the fetus, DA needs to remain open, therefore vasodilating factors are more active than vasoconstrictive factors. Prostaglandins play a dominant role to oppose constriction. Since the pulmonary artery pressure is very high, the pressure within the DA is very high, preventing it from constriction.

### Prostaglandins

**Figure 2:**
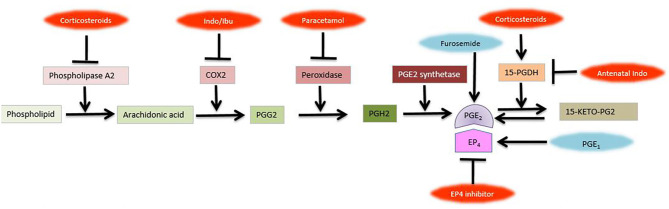
Prostaglandin pathway and metabolism. Inhibitors of various steps are shown in red., COX-1, cyclooxygenase-1; EP4, Prostaglandin receptor 4; Indo, Indomethacin; Ibu, Ibuprofen; PG, prostaglandin; 15-PGDH, 15-hydroxyprostaglandin dehydrogenase; 15-KETO-PG2 (Figure courtesy of Fahri Ovali).

Prostaglandins are synthesized from arachnidonic acid by cyclooxygenases COX1 and COX2 within the ductal muscle. Circulating prostaglandins, originated from the placenta contribute to the patency of DA (Figure 2). Prostaglandins are metabolized in the lung and low pulmonary blood flow in the fetus leads to reduced clearance of prostaglandins, increasing their concentration further.

Prostacylin (PGI_2_) is the major arachnidonic acid product of the ductus but although it is more prevalent, it is less potent than prostaglandin E2 (PGE_2_), which is the most important prostaglandin to regulate the patency of DA (9). PGE_2_ interacts mainly with its receptor EP_4_. EP_4_ activates voltage-gated potassium channel (Kv) and outward K^+^ current increases, thereby inducing membrane hyperpolarization, which inhibits Ca^2+^ influx via the voltage-gated L-type calcium channels (Ca_L_) and decreases intracellular calcium (10). PGE_2_also activates cyclic adenosine monophosphate (cAMP), which inturn activates protein kinase (PKA), which inhibits myosin light chain creatine kinase (MLCK). Inhibition of MLCK blocks phosphorylation of myosin light chains, which results in inhibition of vasoconstriction, hence vasodilation occurs (11–13) (Figure 1). *In utero* vasodilation of the ductus is maintained by dephosphorylation of MLC to MLCP. MLCP is enhanced due to the inhibitory effect of PKG on the Rho-kinase pathway. PKG also activates Kv channels, leading to hyperpolarization and inhibition of Ca^2+^ influx through Ca_L_ channels. Stimulation of sarcoplasmic reticulum calcium ATPase (SERCA), by PKG leads to uptake of Ca^2+^ in sarcoplasmic reticulum, lowering intracellular calcium (14, 15).

### Nitric Oxide

Nitric oxide (NO) is produced by endothelial nitric oxide synthase in the luminal endothelium and endothelium of vasa vasorum and maintains the patency of DA by acting through cGMP. After its production, it diffuses into the SMC and binds with soluble guanylyl cyclase, producing cyclic guanosine monophosphate (cGMP). cGMP activates cGMP-dependent protein kinase (PKG), which induces vasodilation (16) (Figure 1). NO is also formed in small amounts in the SMC by inducible nitric oxide synthase (iNOS) and neuronal NOS, which contribute to ductal relaxation (17). Nitric oxide is used commonly in preterm infants for the management of pulmonary hypertension and may affect the patency of the ductus. PGE_2_ and NO are coupled for reciprocal compensation such that inhibition of one of them increases the concentration of the other one (18). This might explain why some preterm infants fail to close their ductus in response to COX inhibitors.

### Carbon Monoxide

Hem oxygenase 1 and hem oxygenase 2 which produce carbon monoxide (CO) are found in the endothelial and smooth muscle cells of the DA. CO dilates the ductus by inhibition of O_2_ sensing cytochrome P450, thus interrupting endothelin-1 signaling (19, 20) (Figure 1). Carbon monoxide produced under physiological conditions seems to be negligible with regard to ductal patency but in cases of upregulation such as endotoxemia, it may have a relaxing effect on the ductus. Carbon monoxide is also formed in a molar ration during bilirubin production. Theoretically, high bilirubin levels may be associated with ductal patency. However, since high bilirubin levels are not allowed and treated accordingly in human neonates, this effect does not seem to be an important contributor to PDA. Similarly hydrogen sulfide inhibits DA tone (21).

Phosphodiesterase 3 (PDE_3_) inhibitors such as milrinone and amrinone have positive inotrope and vasodilator effects. Inhibition of PDE_3_ also induces dilation of the fetal and postnatal ductus arteriosus in humans. This effect is mediated by an increase in cAMP and cGMP in vascular smooth muscle (22) (Figure 1).

Transient receptor potential melastatin 3 (TRPM3) is expressed heavily in ductal SMC and acts as a calcium channel which increases intracellular Ca^2+^ independent of Ca_L_ channels. Progesterone is a natural inhibitor of TRPM3 and may prevent ductal closure *in utero* (23).

Factors that maintain ductal patency *in utero* are summarized in Table 1 (24).

**Table 1:** Factors that maintain ductal patency *in utero*.

Low oxygen tension
High levels of circulating prostaglandins
Increase in nitric oxide production
Increase in carbon monoxide production
High levels of circulating adenosine
Increase in intracellular cAMP and cGMP
Raised plasma concentrations of atrial natriuretic peptide
Activation of potassium channels Kv1.5, Kv1.2, Kv2.1, KATP, BKca

## Functional Closure

Functional closure of the ductus occurs by 8 h in 44% of normal term infants and almost 100% are closed by 72 h. (25). The rate and degree of this closure is determined by the balance of vasodilator and vasoconstrictive factors. Several changes in late gestation contribute to an increase in ductal tone.

Constriction of the ductus is maintained via3 factors:

a) Decreased concentration of prostaglandins by the loss of placental source and increased removal by the lungs, and decreased number of EP_4_ receptors on the ductal wall.
b) Increased arterial oxygen pressure.
c) Decreased pulmonary vascular resistance resulting in decreased blood pressure within the lumen of the DA.

### Decreased Prostaglandins

After the cord is clamped, PGE_2_ concentrations drop rapidly due to removal of placental production and increased metabolism in the lungs (9). The metabolism of PGE_2_in the lung is mediated by PG transporter, which controls the uptake of PGE_2_into type II alveolar epithelial cells (26). In the alveolar epithelial cell, PGE_2_is degraded reversibly to biologically inactive metabolite 15-keto-PGE_2_ by 15-hydroxyprostaglandin dehydrogenase (15-PGDH) (27). The activity of 15-PGDH increases with increasing gestational age (28). 15-keto-PGE_2_is metabolized irreversibly to 13, 14-dihydro- 15-keto-PGE_2_by Δ-13-reductase. After birth, expression of EP_4_ decreases and increased oxygen reduces the sensitivity of the ductus to PGE_2_ (29). On the other hand, lungs also produce bradykinin (27). Bradykinin induces vasodilation *in utero*, but in higher concentrations, it induces vasoconstriction (30).

In the preterm infant, the sensitivity of DA to PGE_2_ is higher than that in the term infant, which is attributed to increased binding to EP_2_, EP_3_, and EP_4_ receptors (18). The catabolism of PGE_2_ is slower due to low 15-PGDH activity in early gestation. The preterm ductus is also six times more sensitive to dilation to NO than the mature DA (31). Postnatal increase in oxygen might enhance NO production, which induces vasodilation (9).

Although PGE_2_ is the most important agent in maintaining ductal patency in fetal life, there is some evidence that in late gestation, it may paradoxically prepare the ductus for postnatal closure. At that time, PGE_2_ may promote the expression of some genes involved in vasoconstriction (32, 33). In late gestation, some phosphodiesterases that degrade cAMP are upregulated, which results in limited accumulation of cAMP and attenuates the vasodilator effects of PGE_2_ (34).

### Increased Oxygen

There are a number of oxygen sensing tissues in human body, such as fetoplacental arteries in the placenta, pulmonary artery, DA, adrenomedullary chromaffin cells, neuroepithelial body and carotid body. This oxygen-sensing and responding system is called the Homeostatic Oxygen Sensing System (HOSS) and should be considered one of the body's major systems such as cardiovascular, nervous or endocrine systems (35).

During fetal life, oxygen pressure in the ductal lumen is about 18–28 mmHg, which is suitable for maintaining the ductus open (36). After birth, arterial oxygen rises sharply to 80–100 mmHg, which leads to vasoconstriction of almost all vascular smooth muscle, with the exception of pulmonary artery (37). Oxygen-induced constriction begins within 4.6 ± 1.2 min after a rise in PaO_2_ (38).

Oxygen-induced vasoconstriction is a multistep process. Oxygen sensing involves a change in redox state, determined by mitochondria and nicotinamide adenine dinucleonide phosphate (NADPH) oxidase. Proximal electron transport chain is the major oxygen sensor in the ductal SMC. This is reflected in the observation that electron transport chain inhibitors (i.e., rotenone and antimycin) mimic hypoxia and constrict the ductus, as well as activating the carotid body. When PO_2_ rises, mitochondria respond by production of reactive O_2_ species (ROS) especially H_2_O_2_, which changes the cellular redox potential and alters the function of redox-sensitive genes, second messenger systems and oxygen-sensitive potassium channels, which control SMC membrane potential. Production of adenosine triphosphate (ATP) through oxidative phosphorylation increases with rising oxygen levels and inhibits K_ATP_ channels (39). These channels compose of many subunits such as Kv1.2, Kv1.5, Kv2.1, Kv31b, Kv4.3, Kv9.3, and BKca (40).

Response to oxygen is mediated through potassium channels. Although the pulmonary artery and the ductus are continuous and have similar Kv channels, their response to oxygen is reversed. Hypoxia causes pulmonary artery vasoconstriction and ductal vasodilation. Reduced expression of Kv1.5 and Kv2.1 channels results in failure of ductal closure. It may be speculated that the failed response of premature infants' ductus to normoxia may be related to “deficient” Kv channels, reduced PGE_2_induced gene expression of ion channels (41) and a gene transfer which restores K2.1 or Kv1.5 expression might be helpful for vasoconstriction (40).

Inhibition of potassium channels leads to membrane depolarization, influx of calcium into the cells through L type (Ca_L_) and T type (Ca_T_) calcium channels, and opening of inositol triposphate (IP3) sensitive sarcoplasmic reticulum calcium stores which release calcium (11, 42). This causes DA constriction without depolarization. Subsequent calcium entry through these channels promotes constriction of the DA.

L-type calcium channels are themselves oxygen-sensitive (43). T-type calcium channels also regulate calcium entry into the cells and they are upregulated with increased oxygenation (44). In response to oxygen, calcium may also enter the cell through reverse-mode function of the Na/Ca exchanger also. Maturation of the sensor, mediator and effectors goes in parallel (45).

Cytochrome P450 enzymes catalyze a number of reactions to modulate inflammation, angiogenesis and vascular tone. A member of this system also acts as a sensor for oxygen (46). Monooxygenase and lipooxygenase metabolites of arachidonic acid have the ability to respond to changes in oxygen tension (47). Stimulation of the endothelin A receptor and production of endothelin-1 (ET-1) mediates ductal constriction (48).

Isoprostanes are prostaglandin-like compounds which are produced in response to oxidative stress via non-enzymatically free radical mediated peroxidation of arachidonic acid, without the effect of COX enzymes. They increase as a response to increased arterial PO_2_ and increased ROS and mediate inflammation as well as constriction of the DA (49).

Retinoic acid is also active in oxygen signaling and maternally administered vitamin A accelerates the development of oxygen sensing mechanism by increasing the expression of Cav1.2 and Cav3.1 in the rat DA (50).

Rho-kinase pathway is effective in sustaining the contractile state of the ductus. Increased ROS, mainly H_2_O_2_ in the mitochondria induces RhoB and Rho-associated protein kinase-1 (ROCK-1) expression, which promotes phosphorylation of myosin phosphatase inhibiting MLCP. Activation of the Rho-kinase pathway induces calcium sensitization, which sustains ductal constriction through positive feedback mechanism (51). Rho-kinase system balances MLCK and MLCP (Figure 1). Inhibitors of Rho-kinase such as fasudil lead to pulmonary vascular relaxation, thus pulmonary hypertension (52). In the preterm infant, mitochondrial ROS system is immature, and together with failure to upregulate Rho-kinase expression to oxygen, leads to ductal vasodilation. Exogenous H_2_O_2_ mimics the effects of increased oxygen.

Oxygen stimulates the release and synthesis of endothelin-1 (ET-1), acting on type A receptors (ET_A_). Stimulation of these receptors induces IP_3_ production by phospholipase c, which leads to an increase in intracellular Ca^2+^ (53). Endothelin receptor antagonists are found to inhibit ductal constriction (54).

In the preterm infant smooth muscle myosin isoforms are immature and the contractile capacity of the muscle cells are weak. L-type calcium channels are immature with impaired calcium entry into the cells. Reduced expression of Rho kinase expression and activity as well as oxygen sensing KV channels contribute to the ductus patency (45). Inhibition of these potassium channels by oxygen leads to SMC depolarization, opening of the voltage-gated L-type Ca channels, influx of calcium into the SMC and vasoconstriction. The preterm ductus has increased sensitivity to the vasodilating effects of prostaglandins and nitric oxide, which prevents constriction. This sensitivity is affected by increased cAMP signaling and decreased cAMP degradation by phosphodiesterases (55). High rates of response to PG inhibitors such as indomethacin and ibuprofen may be explained by this exaggerated sensitivity of the ductus to PGs. On the contrary, elevated levels of PGs during sepsis or necrotizing enterocolitis may mediate the re-opening of ductus in preterm infants (56).

### Non-oxygen Pathways

In the preterm ductus energy metabolism begins to fall after birth, with decreased amounts of glucose, oxygen and adenosine triphosphate (ATP). This is not profound enough to cause cell death but interferes with the ability of the ductus to close (57).

Glutamate promotes ductal constriction by glutamate inotropic receptor subunit 1 (GluR1)-mediated noradrenalin production in animal models (58). It may be suggested that nutritional supply of glutamate may have a therapeutic role in the closure of PDA (59).

Caffeine is an adenosine receptor blocker, but it also affects several mechanisms involved in ductal closure. It increases cAMP, releases Ca^+2^ from endoplasmic reticulum, and inhibits the production and activity of prostaglandins. It is used frequently in extremely low birth weight infants for the prevention and treatment of apnea and bronchopulmonary dysplasia. However, it does not affect the contractile response of the ductus at therapeutic concentrations (60).

There is a transient decline in serum osmolarity after birth, which recovers to adult levels within a few days There is some evidence that hypo-osmolarity may mediate ductal closure. In preterm infants with hemodynamically significant PDA, osmolarity is 5 mOsm/L higher compared to non-hemodynamically significant PDA group (61). Hypo-osmotic receptor transient receptor potential melastatin 3 (TRPM3) is upregulated in the ductus and regulates calcium influx into the cells, promoting vasoconstriction. Rats with transient hypo-osmolarity after birth have increased rates of ductal closure (62).

B-type Natriuretic peptide (BNP) plays an important role in regulating the body fluid volume and blood pressure. It is secreted from the ventricles in response to increased cardiac stress and increases intracellular cGMP, inducing vasodilation. In PDA, the volume of left-to-right shunting is associated with the BNP level and higher levels predict a hemodynamically significant PDA and a poor response to indomethacinin preterm infants (63, 64). Bradykinin induces ductal constriction through BK-1 receptors (29).

### Progesterone

Progesterone regulates prostaglandin synthesis and prostaglandin sensitivity of smooth muscle cells. Progesterone withdrawal in late pregnancy coincides with the increasing sensitivity of ductus arteriosus to constriction by indomethacin (65). This effect is similar to that of glucocorticoids (66). However, this effect seems to be pharmacological, rather than physiological (67).

In summary, the overall mechanism of functional closure of the ductus involves a drop in PGE_2_ levels after birth, which impairs ductal vasodilation and a concomitant rise in oxygen which leads to vasoconstriction. Intracellular calcium increases, secondary to inhibition of Kv channels and K_ATP_ induced membrane depolarization. Increased calcium within the cell activates Ca^2+^–Calmodulin dependent MLCK activation, which phosphorylates MLC (8). Calmodulin expression is upregulated after birth (68). Increased ET-1 synthesis releases intracellular calcium from the sarcoplasmic reticulum (69). Rho-kinase pathway inhibits MLCP, inducing calcium sensitization by reducing the requirement for calcium influx, maintaining vasoconstriction (9).

Factors that contribute to ductal constriction after birth are summarized in Table 2 (23).

**Table 2:** Factors that contribute to ductal constriction after birth.

High levels of oxygen
Low levels of circulating prostaglandins
Increase in endothelin-1
Activation of cytochrome P450
Rise in intracellular calcium
Decrease in intracellular cAMP and cGMP
Inhibition of potassium channels
Production of isoprostanes
Response to acethycholine
Response to norepinephrine
Activation of transient receptor potential channels
Activation of Rho-kinase family members
Release of angiotensin II

## Anatomic Closure and Remodeling

The initial constriction of the ductus alone is insufficient for the permanent cessation of blood flow through it. Permanent anatomic closure is essential to prevent re-opening, which occurs through a process of remodeling. This process involves intimal cushion formation, disassembly of internal elastic lamina and loss of elastic fibers in the medial layer, migration and proliferation of the SMCs, extracellular matrix production and endothelial cell proliferation and finally blood cell interaction. Each step is related to each other and occurs in a sequential way. After complete anatomic closure, the ductus undergoes apoptosis and becomes the ligamentum arteriosum.

### Vasa Vasorum

**Figure 3:**
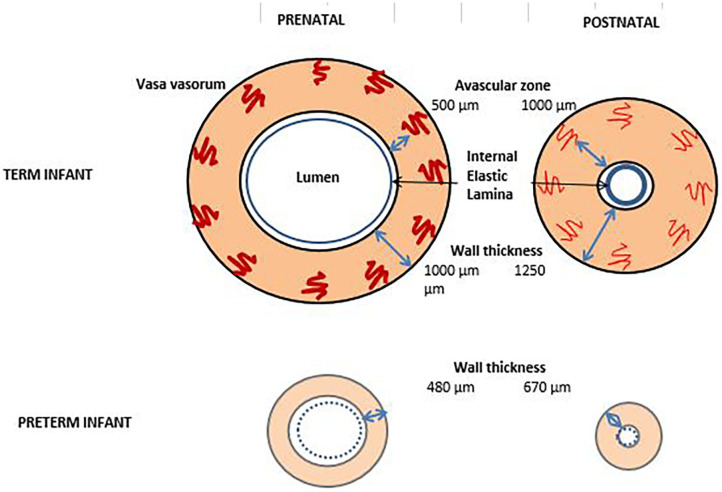
Vaso vasorum in term and preterm infants before and after birth. By the obliteration of vaso vasorum, the thickness of the vaso vasorum increases after birth and the lumen shrinks. In the preterm infant, vaso vasorum are absent or very scarce and the elastic lamina is thin [Figure adapted from (72)].

A substantial amount of nutrients of the ductal wall is provided by vasa vasorum, which supply the outer portion of the wall. Vasa vasorum enters the outer wall of the ductus and grow toward the lumen. They stop growing ~400–500 μm from the lumen. The distance between the lumen and the tip of vasa vasorum is called the avascular zone of the vessel. The thickness of the avascular zone defines the furthest distance that still maintain oxygen and nutrient homeostasis in the tissue. Vasa vasorums appear in the ductal wall only after wall thickness exceeds 400 μm which is not evident before 28 weeks of gestation (70, 71). The thickness of the ductal wall in the full term fetus is 1,000 μm. When the ductus constricts after birth, it increases up to 1,250 μm, in lieu of the lumen (9). In the preterm fetal ductus, the wall thickness is about 480 μm and it increases to 670 μm after birth, when constricted (9) (Figure 3). In the full-term ductus, the increased tissue pressure occurring during ductus constriction occludes the vasa vasorum and prevents flow of nutrients to the outer wall of the vessel. This results in the effective avascular zone expanding from 500 μm to the entire thickness of the vessel wall. When this occurs, the center of the ductus wall becomes profoundly ischemic (73). However, in the 24-week-old preterm infant, the ductus is only 200 μm thick. The vasa vasorum do not penetrate the muscle media. This thin walled ductus does not need the vasa vasorum for nutrient flow. As a result, there is no vulnerable region of the wall during closure of the ductus. The preterm ductus arteriosus is less likely to develop the severe degree of hypoxia that is necessary for ductal remodeling (74).

In the term infant, increased intramural pressure occludes vasa vasorum leading to the inhibition of provision of nutrients and oxygen to the muscular layer. Thereafter, the blood and hence the oxygen supply to the cellular layer subsides, leading to hypoxia and cell death (73). The profound hypoxia that follows induces local production of hypoxia inducible factors 1α and VEGF, inhibits the production of PGE_2_ and results in apoptosis of SMCs. The proliferation of endothelial cells is mediated by VEGF. Adhesion of mononuclear cells is mediated by VEGF whereas the formation of the platelet plug is mediated by platelet-derived growth factor. This induces an inflammatory response and monocytes and macrophages begin to recruit to the ductal wall, producing platelet-derived growth factor which is essential for migration and proliferation. During the initial functional vasoconstriction, loss of luminal blood flow causes hypoxia in the ductal muscle, inducing VEGF secretion (74).

Since the ductal vessel wall is very thin in the preterm infant, it does not contain vasa vasorum and the cells receive oxygen by diffusion from the lumen. This means that as long as luminal patency continues, the cells will receive oxygen and will fail to undergo anatomic remodeling. The preterm ductus requires a greater degree of constriction than that of the term infant in order to achieve a similar degree of hypoxia to trigger the cascade of events necessary for anatomic closure. At the same time, they will be responsive to prosglandins, and be susceptible to re-opening in case of prostaglandin surge as it happens in sepsis or necrotizing enterocolitis. In contrast, preterm ductus produces NO after birth, due to the growth of new vasa vasorum that synthesize NO (63). Therefore, the ductus becomes more dependent on vasodilators other than prostaglandins after the first few weeks. This is reflected in the decreased response to indomethacin with increasing postnatal age. In animal data, combined use of NO-synthase inhibitors with indomethacin produces a greater ductus constriction than indomethacin alone (75).

### Elastic Fibers

The structure of the ductal wall is strikingly different from the aortic wall with regard to its elastic content. In great arteries, complex extracellular matrix and elastic fibers are essential to overcome the mechanical and hemodynamic stress of the pulsatile flow. Well-developed elastic fibers in the aorta prevents it from collapsing. However, in the ductal wall, there are very few and thin elastic fibers which do not prevent, but in fact facilitate ductal collapse. Ductal wall is composed of just a media layer which consists SMCs and a superficial thin internal elastic lamina (Figure 3). This lamina is also very prone to fragmentation. The intima layer contains the endothelial cells facing the lumen of the ductus. The structural features of the ductus prepares it for immediate closure after birth. Abnormalities of the elastic fibers and elastic lamina may be responsible for some of the PDA cases by preventing intimal cushion formation and collapse of the ductal wall (73).

In the ductal wall, there is less elastin binding protein leading to less elastin deposition. Moreover, increased chondroitin sulfate in the ductus leads to dissociation of tropoelastin from the cell surface, interfering with elastin fiber assembly further (76). Tropoelastin is a chemoattractant for SMCs. Lysl oxidase (LOX) catalyzes elastin cross-linking and it is degraded in the ductal cell when EP_4_ receptor is stimulated by the prostaglandins (77). EP_4_ is a Gs protein coupled receptor which increases intracellular cAMP by adenylyl cyclases. Signaling coupled with PGE_2_ and EP_4_ stimulates vascular dilation and intimal thickening.

### Intimal Cushion Formation

After the functional phase, progressive intimal thickening and fragmentation of the internal elastic lamina occurs, ultimately forming protrusions which occlude the ductal lumen. During the remodeling process; endothelial cells and internal elastic lamina separate, leaving a subendothelial space in-between for the migration of SMCs and endothelial cells. Integrins, which are transmembrane receptors on the cell surface participate in the interaction of these cells, promoting Intimal cushion formation. In patients whose ductus is not closed, integrin expression is downregulated (78). Intimal thickening is due to the migration of smooth muscle cells from the media layer to the intima and to the proliferation of luminal endothelial cells. During this process, the first step is the migration of SMC from the media to the subendothelial layer, leading to neointimal cushion formation (79). SMC migration is mediated by PGE_2_, Transforming Growth Factor Beta (TGFβ-1), notch signaling, Vascular Endothelial Growth Factor (VEGF) and fibronectin coupled with hyaluronan. The proliferation of SMCs is mediated by retinoic acid and notch signaling. Mounds are formed by expansion of the neointima by hyaluronan, which creates a suitable space for migrating SMC from the muscle into the intima and proliferating endothelial cells. The process begins with the accumulation of hyaluronan beneath the endothelial cells, accompanied by loss of laminin and type IV collagen and their separation from the internal elastic lamina. Influx of water into the hyaluronan widens the subendothelial space. Hyaluronan potentiates the migration of SMCs to the subendothelial space through hyaluronan binding protein, synthesized by SMCs (80). This wide space is suitable for SMC migration. Smooth muscle migration is facilitated by impaired elastin assembly and elastin fragmentation. Accumulation of hyaluronan is associated with increased production of TGFβ and prostaglandins via activation of adenylylcyclase 6 (81). Ductal SMCs also secrete fibronectin and chondroitin sulfate which facilitate the migration of these cells (82). SMC also secrete laminin, which is a promigratory protein (83).

In addition to its effects on vasodilation, PGE_2_ stimulates SMC migration through exchange protein activated cAMP (epac) pathway in the ductal wall (84) (Figure 1). Cyclic AMP has 2 targets: protein kinase A (PKA) and epac. Epac is a guanine nucleotide exchange protein independent of PKA, which regulates the activity of small G proteins. It is upregulated during the perinatal period and promotes SMC migration without hyaluronan production (85). cAMP is degraded by phosphodiesterases (PDE) to 5'AMP. Activation of PDE can suppress cAMP mediated EP_4_ signaling and PDE inhibitors can enhance it (12). Neointimal mounds are less well-developed in the preterm infant, rendering the occlusion of the lumen difficult.

### Retinoic Acid

Retinoic acid stimulates the growth of SMCs and decreases apoptosis (86). Prenatal administration of vitamin A increases the production of fibronectin and hyaluronic acid, leading to intimal tickening, as well as the intracellular calcium response and the contractile response of the ductus to oxygen (50). This response is activated by the upregulation of α1G subunit of voltage-dependent calcium channel, which is activated by oxygen-induced inhibition of potassium channel (32). In other words, retinoic acid stimulates both functional and anatomic closure of the ductus. Early trials in human neonates concluded that administration of vitamin A after birth does not result in constriction of DA, but retinoic acid receptor activation may be have a therapeutic potential for treating PDA (87, 88). However, in a recent trial on preterm infants administering 10,000 units of Vitamin A on alternate days for 28 days, rate of PDA was significantly reduced (89).

### TGF-β1

TGF-β1 binds the SMC through integrins to the extracellular matrix, slowing down the migration. This is necessary during the remodeling process for maintaining the tension to sustain DA contraction (90).

### Interleukin-15

(IL-15) has many pro-inflammatory effects, but also inhibits SMC proliferation and hyaluronan accumulation during the remodeling process of the ductus. The up-regulation of IL-15 in the ductus is mediated through the prostaglandin pathway. IL-15 upregulates the expression of EP_4_ mRNA, as well as stimulating angiogenesis through binding to endothelial cells (91). In late gestation, several factors try to promote the structural closure of the ductus in preparation for the postnatal life and IL-15 may balance this tendency by a counteractive mechanism (92).

### Notch Signaling

Notch receptors are critical for cell differentiation and there are 4 Notch receptors in humans. Notch 2 and Notch3 receptors are abundant in smooth muscle and they regulate proliferation, migration and angiogenesis in the vasculature. A decrease in the number of Notch receptors in SMCs is associated with downregulation of gene expressions related to contractile elements (84). Notch signaling is required for contractile SMC differentiation in mice (91).

### Extracellular Matrix Production and Proliferation

Extracellular matrix consists of hyaluronan, fibronectin, chondroitin sulfate and elastin, which are mediated by retinoic acid, TGFβ, PGE_2_, IL-15, and oxygen (51).

Hyaluronan is effective in SMC migration and regulated by PGE_2_, IL-15, and TGFβ. PGE_2_-mediated EP_4_ activation increases the cAMP production through protein kinase A (PKA), which stimulates hyaluronan production in the SMCs (93).

Fibronectin is secreted by the SMCs and promotes SMC migration in the process of intimal cushion formation. Ductal SMC produce twice more fibronectin than aortic SMCs (94). It is possible that preventing fibronectin dependent intimal thickening may be utilized for the patency of DA in congenital heart diseases (82). Other mediators which play an important role in the proliferation and migration of SMCs include versican, a hyaluronan-binding proteoglycan and tanacin, a hexameric glycoprotein (95, 96).

Chondroitin sulfate causes the release of elastin binding protein from the SMCs, impairing the elastin assembly and stabilizing the hyaluronan. Therefore, it indirectly promotes the migration of SMCs and upregulates the synthesis of fibronectin (97).

Elastin contributes to the patency of the ductus by maintaining the elasticity of the ductal wall. Its production is regulated by oxygen and PGE_2_. Inhibition of elastogenesis by PGE_2_ causes vascular collapse and ductal closure. Oxygen reduces elastin secretion in the SMCs. Stated otherwise, PGE_2_ and oxygen stimulates elastogenesis, leading to vasodilation and patency of the ductus (72, 98).

### Blood Cell Interactions

Vascular wall ischemia induces a local inflammatory response, which activates mononuclear cells in the blood. Leukocyte interactions with P-selectin and Intercellular Adhesion Molecule-1 (ICAM-1) is necessary for recruitment when physiologic shear forces are present and this interaction is only possible when the flow is very slow. Therefore, leukocytes can adhere to the ductal wall only after vasoconstriction and loss of ductal flow has occurred (99, 100) Monocytes and phagocytes adhere to the wall of the ductus via vascular cell adhesion molecule-1 (VCAM-1), whose expression increase after birth (7, 91). VEGF is a direct chemoattractant for monocytes (101). The magnitude of this adhesion is correlated by the extent of intimal cushion formation (102). Monocytes also stimulate PDGF-B, which is a mediator of smooth muscle migration (103).

During the initial process of vasoconstriction, detachment of endothelial cells triggers the adhesion of platelets to the “injured” site, forming a platelet plug, which further blocks the lumen. Mice with disrupted platelet function or administered antibody against platelets have impaired ductus remodeling (104). The effect of platelets on ductal closure is minimal in term infants but it is more pronounced in preterm infants. Since the intimal cushion formation is mainly triggered by hypoxia and the preterm ductus is less likely to become hypoxic when it constricts after birth, platelets play a more significant role in ductal plug formation and closure in this setting (105). It is reported that platelet-derived growth factor is lower in infants with PDA (106). Low platelet count is also correlated with hemodynamically significant PDA and delayed closure of the ductus (104, 107), whereas high platelet counts promote the initial constriction of the ductus (108).

## Genetic Background

Although they originate from the same neural crest lineage, there are major transcriptional differences between the DA and aorta. Several studies highlight the effect of genetic predisposition to the patency of DA. These include either the prostaglandin pathway (i.e., smooth muscle contraction), or vascular smooth muscle development or structure. Most of these information is obtained through mouse models. Overall, there are more than 100 genes are effective in the development of DA (33, 109).

Arachidonic acid is metabolized by PGH_2_ synthase to prostaglandin H_2_, which in turn is metabolized by PGE synthase to prostaglandin E_2_ (PGE_2_). Prostaglandin endoperoxide synthase 1 (*Ptgs1* or *COX1*) and (*Ptgs2* or *COX2*) is the rate limiting enzyme in the production of prostaglandins from arachidonic acid. Mice deficient in *Ptgs1* have normal survival after birth whereas 35% of mice deficient in *Ptgs2* die shortly after birth because of PDA (110). If both are lacking, 100% of mice die in the first day (111). Prostaglandin E_2_ binds to the cell through its receptor EP_4_ (*Ptger4*) and mediates vascular smooth muscle relaxation and intimal thickening. Mice lacking these receptors die due to PDA (11). Furthermore, *Hpgd* gene encodes hydroxyprostaglandin dehydrogenase 15-(NAD) which is responsible for PGE_2_ metabolism. The deletion of this gene leads to elevated levels of PGE, leaving the DA open and resulting in death (112). Mice deficient in prostaglandin transporter gene Slco2a1 also die within 2 days after birth (113).

The effects of prostaglandins are mediated through cyclic guanosine monophosphate (cGMP) and cyclic adenosine monophosphate (cAMP). Phosphodiesterases degrade these compounds and several genes have been proposed for phosphodiesterases. *Pde5a* mediates vasoconstriction in SMC of DA and its expression decreases in 6 h after birth (114).

The genes upregulated during ductal closure are phosphodiesterase 4B (PDE4B), desmin pyruvate dehydrogenase kinase isozyme 4 (PDK4), and insulin-like growth factor binding protein 3 (IGFBP3). Downregulated genes include cyclin-dependent kinase inhibitor 1C (CDKN1C) and alpha-2-glycoprotein1 (AZGP1). PDE4B limits the accumulation of cAMP, minimizing the vasodilator effect of endogenous prostaglandins. Therefore, increasing PDE4B would facilitate ductal constriction. Desmin is a component of the cytoskeleton and its increase indicates the maturation of SMCs. PDK4 and IGFBP3 regulate cell proliferation and apoptosis, which are operative in ductal closure. Increased transforming growth factor β-receptor II regulates PDK4 also (90, 115, 116). Wingless integrin pathway (Wnt), which is a part of IGFBP3 system plays a major role in ductal closure through upregulation of cyclooxygenase 2 and apoptotic processes mediated by macrophages (117).

Smooth muscle of the DA contracts in response to oxygen. Mice deficient in myosin heavy chain gene (*Myh11*) have delayed closure of DA (118). Myocardin regulates expression of genes for contractile proteins in smooth muscle cells (SMC) and selective deletion or ablation of *Mycocd* results in death (119). *Tcfap2b* is the gene which encodes the transcription factor in neural crest cells, from which the DA originates. Mice deficient in *Tcfap2b* die within the first day of life with PDA and pulmonary overload (120). The DA of these mice lack markers of differentiated SMCs such as calponin and hypoxia-inducible factor 2α (HIF2α) which are also involved in oxygen sensing, suggesting that *Tcfap2b* plays an important role in the development of SMCs (121). Some genes act together resulting in PDA. Whereas, the deletion of *Notch2* gene results in PDA in only 40% of cases, when combined with the deletion of *Notch3*, PDA is observed in 100% of cases (122). Deletion of the *Notch* ligand, *jag1* also results in lethal PDA on day 1 because of significant defects in SMC differentiation (123). On the other hand, deletion of the Brahma-related gene (Brg1-a) which is responsible for chromatin remodeling results in cardiovascular anomalies, including PDA (124). Genes involved in platelet production or adhesion such as *Nfe2* or *Itga2b* affect the complete occlusion and remodeling of the DA (104).

Changes in the expression of structural genes also affect the patency of DA. Vascular SMC contraction is mediated by myosin II. Myosin II has two components, namely heavy chains and light chains. The genes encoding these proteins such as *Myh11, Myl2, Myl5, Myl7*, and *Myl9* may be effective in the regulation of ductal patency, but more studies are needed to reach a definite conclusion (68, 125). Contraction of the myosin light chains requires phosphorylation and this step is regulated by the Rho-Rho kinase system. The gene encoding the RhoBGTPase (*Rhob*) and its effector *Rock2* is upregulated in the newborn DA and inhibition of this system may result in the patency of DA (38).

L-type calcium channels are essential for ductal closure and the genes encoding the Na/K ATPase (*Akq1b1*) and a subunit of L-type calcium channels (*Cacna2d1*) are highly expressed in the DA (126). Potassium channels control the resting membrane potential of SMCs and the genes encoding these channels (*Kcnk3* and *Abcc9*) are also upregulated in the DA (127). Large conductance calcium-activated potassium channels (*BKca*) are abundant in vascular smooth muscle and they are expressed significantly higher in DA (109). These ion channels may be promising targets for novel PDA therapies.

Vasoconstrictive effect of oxygen in the DA is mediated by endothelin-1 (ET-1). There are 2 membrane receptors for ET-1, ET_A_ receptor is encoded by the *Ednra* gene and ET_B_ receptor is encoded by the *Ednrb* gene. ET_B_ receptor functions as a scavenger for ET-1 and inhibits endothelin dependent vasoconstriction. Downregulation of this receptor would result in increased levels of ET-1, leading to vasoconstriction of the ductus, which takes place in late preterm infants (68, 126).

In humans, there are a number of syndromes associated with PDA, as well as non-syndromic PDA which are associated with single nucleotide polymorphisms (128). In twin studies, a familial component has been suggested for PDA in preterm infants (129). Consanguineous marriages provide insights to specific chromosome regions associated with increased susceptibility to PDA (130). A polymorphism in the estrogen receptor alpha gene ESR1 *rs2234693*, prostaglandin I2 synthase gene *(rs493694)* and another polymorphism in the interferon gamma gene *rs2430561* is associated with a decreased risk of PDA and bronchopulmonary dysplasia (131, 132). On the other hand, polymorphisms in the TNF receptor associated factor 1 TRAF1 (*rs1056567*) gene, angiotensin II type 1 receptor AGTR1 gene and TFAP2β (*rs987237*) gene, are associated with increased risk of PDA (133, 134). TFAP2β is also associated with altered levels of calcium and potassium channels in SMCs, which are essential for proper contraction (135). Angiotensin II is vasoconstrictive for the ductus and its receptor (*Agtr2*) is upregulated in late preterm infants also (128).

Although their significance remain to be elucidated, there are other genes involved in the development of matrix molecules of the DA, such as *Tcfab2b*, lysyl oxidase (*Lox*), and fibronectin (*Fn1*) genes which may draw attention in the future (128). Lysyl oxidase is a cross-linking enzyme for elastic fibers and stabilizes the extracellular matrix. Reduction of LOX activity is demonstrated in the pathogenesis of many vascular diseases (136). Activation of PGE_2_-EP_4_ pathway promotes the degradation of this enzyme, leading to poor elastogenesis, suggesting that control of elastogenesis is clinically important (72). Paradoxically, there are some data that indomethacin treatment of preterm infants may have an adverse effect on the inhibition of elastic fiber formation (72). Regulation of the LOX protein through PGE_2_-EP_4_ pathway may be a basis for further therapeutic strategies that target vascular elastogenesis (72).

## Mechanical and Hemodynamical Factors

### Hemodynamic Changes After Birth

In fetal life, high pulmonary arterial pressure, coupled with low systemic resistance in the placenta results in right to left shunting of blood through the ductus so that a large proportion of blood coming from the umbilical circulation is directed to the systemic circulation without passing through the pulmonary vascular bed. During fetal life, 10–20% of total biventricular cardiac output enters the lungs but in late gestation, this increases to 30% (137). After birth, umbilical cord clamping increases the systemic resistance whereas ventilation of the lungs decreases the pulmonary vascular resistance and increases the pulmonary blood flow; the shunt through the ductus reverses to left to right. This sudden reversal of shunting across the DA rises the pulmonary blood flow even further. In term infant, dominant left to right shunting is achieved by 10 min after birth and it is entirely left to right by 24 h of age (138). In critical cyanotic congenital heart disease, patency of ductus is life-saving. In persistent pulmonary hypertension, high resistance in pulmonary arteries results in persistance of right to left shunting through the ductus. In the preterm neonate, if the ductus remains open, left to right shunting will occur both during systole and diastole and large amounts of blood would flood the lungs. This will expose the endothelium to increased shearing forces, which will induce the production of vasodilatory mediators such as nitric oxide, bradykinin, prostacyclin, and inactivate the production of vasoconstrictor mediators such as thromboxane, endothelin and leukotriens. Arachnidonic acid may be released from pulmonary tissue damaged by shear stress, secondary to mechanical ventilation. Prolonged mechanical ventilation can also induce the release of prostacyclin, often associated with increased ductal shunting (139). This would lead to alveolar edema, and increased need for respiratory support. Positive pressure ventilation during resuscitation, oxygen, and exogenous surfactant treatment induce a rapid fall in PVR. Especially in preterm infants <32 weeks, surfactant administration is associated with an increase in the right ventricular output and absolute ductal diameter (140, 141). Acute rise in pulmonary blood low causes a significant increase in left heart preload and rise in left atrial pressure which results in increased pressure in the left atrium and contraction of foramen ovale (142). The myocardium of the preterm infant is less contractile due to fewer contractile units, increased water content and immature sarcoplasmic reticulum (143). Thus, hemodynamic stability of the preterm infant is highly dependent on myocardial function, and a sudden increase in the workload of myocardium after birth may result in failure of the myocardium resulting in pulmonary overload and hypertension. In preterm infants, delayed cord clamping does not seem to affect the incidence of PDA (144). Early adrenal insufficiency may also have a role in prolonged ductal patency (145).

Fluid dynamics state that the shunt volume across a tubular structure is directly proportional to the 4th power of the radius of the tube (i.e., ductus) and the pressure gradient between both ends (i.e., aorta and pulmonary artery), and inversely proportional to the blood viscosity and length of the tube (i.e., ductus). Moreover, increased venous return from the lungs to the left ventricle will cause an increase in the left ventricular end-diastolic pressure, which will increase atrial natriuretic peptide (ANP), with evolution of pulmonary venous hypertension and pulmonary hemorrhage. On the other hand, left to right shunting at the ductus will decrease the systemic perfusion (i.e., ductal steal) leading to systemic hypoxia and organ dysfunction. Shorter diastolic time and increased myocardial oxygen demand may result in endocardial ischemia (142).

There is scarce information on the effects of dilation mediated by the mechanical force of the flow, or through shear forces and shear stress. Although there is no clear evidence, it may be speculated that there are some mechanosensors in the ductal wall, which can sense the biomechanical changes in the lumen and regulate ductal closure. The effect of laminar flow vs. turbulent flow across the ductus is also understudied. In case of laminar flow, endothelial cells are less likely to proliferate and nitric oxide synthase and prostacyclin are upregulated (146, 147). However, if turbulent flow develops, endothelial cells begin to upregulate vasoconstrictors such as endothelin-1 and adhesion molecules such as VCAM-1 and ICAM-1 (148, 149). Matrix modeling is an essential component of intimal cushion formation. Stretch of the ductal wall induces the release of angiotensin II from the endothelial cell, leading to smooth muscle proliferation and production of matrix metalloproteinases which trigger extracellular matrix remodeling and vasoconstriction (149, 150). Circumferential stretch also triggers phenotypic switching of SMCs from contractile to synthetic cells, which means more proliferation and migration of SMCs (150).

### Fluid Load

A sharp decrease in pulmonary artery pressure, as it happens after surfactant replacement therapy may increase the left-to-right shunt through the ductus, leading to pulmonary overflow and hemorrhage. In preterm infants, increased pulmonary fluid and protein load is eliminated by an increased lung lymph flow, named as “edema safety factor.” Therefore, ductal closure within the first 24 h of birth do not have any significant effect on the course of respiratory distress syndrome. However, if ductus remains open for several days, this compensation cannot be maintained and alteration in the pulmonary mechanics and pulmonary edema ensue. The impact of patent ductus arteriosus on patient outcomes seems to be a function of magnitude of shunt, rather than its mere presence. Closure of the ductus improves lung mechanics in these infants (151). Early ductal closure is associated with less pulmonary hemorrhages in preterm infants (152). Ibuprofen leads to increased expression of sodium channels and increased removal of fluid from the alveolar compartment (153). In some studies, fluid restriction is recommended as an alternative to treatment; such an approach may reduce pulmonary circulation but also compromises end organ flow in patients already suffering from ductal steal phenomenon (154). However, high (liberal) fluid intake is associated with an increased risk of PDA and BPD (155). Therefore, close monitoring of fluid intake and individualized treatment of infants are of utmost importance in preterm infants, especially in the first days of life.

### Early Adrenal Function

Cortisol is central to the ability of the infant to attenuate its response to inflammation and is potent inhibitor of inflammatory edema. Animal and human studies have shown that adrenal insufficiency can lead to increased inflammatory response to injury and that glucocorticoids can affect patency of the ductus (156, 157). Glucocorticoids induce production of lipocortin which inhibits phospholipase A_2_. Phospholipase A_2_ stimulates the release of arachnidonic acid precursors needed for prostaglandin synthesis; thus in response to steroids, prostaglandin production is inhibited (158). Animal studies have shown that glucocorticoid administration decreases the sensitivity of ductal tissue to the dilating influence of PGE_2_ (156). Sick infants do not have elevated serum corticol levels compared to well infants. These infants are also prone to developing bronchopulmonary dysplasia (159). Therefore, it is not surprising that patent ductus arteriosus is one of the most prominent risk factors in the development of bronchopulmonary dysplasia (160).

### Effects of Surgical Ligation

Surgical ligation of the ductal immediately after birth increases the expression of genes involved in lung inflammation and decreases the expression of genes involved in epithelial sodium channels (161). Therefore, the pulmonary mechanics do not improve as much as expected. Furthermore, early ligation may slow down lung growth (162). On the other hand, in the presence of atelectasis, the persistence of left to right shunt through the PDA maintains an elevated arterial oxygen pressure in the lungs and after PDA ligation, a decrease in the systemic arterial pressure may be observed (163).

## Effects of Pharmacological Agents

Oxygen is the most powerful agent for closure of the ductus. In numerous studies investigating the effect of different levels of oxygen on preterm morbidities, ductal closure was associated with high levels of oxygen but oxygen-related co-morbidities in these infants raised significant concerns (164). These trials including Surfactant, Positive Pressure, Pulse Oxymetry Randomized Trial (SUPPORT), Canadian Oxygen Trial (COT), and Benefits of Oxygen Saturation Targeting (BOOST) II trials have reported neurodevelopmental outcomes after various oxygen targeting, as well as other complications; however the discussion of these trials are out of the context of this article (165–167).

All available pharmacological treatments aiming for changing the ductal tone use the prostaglandin pathway by either the cyclooxygenase pathway (indomethacin and ibuprofen) or the peroxidase pathway (acetaminophen). The infant's gestational age, as well as the infant's race, ethnicity, growth, and exposure to antenatal corticosteroids modify the effects of prostaglandins and determine the rate of drug-induced closure (168, 169).

In the term infant PGE_2_ receptors are down-regulated after birth. However, in the preterm infant, the production of receptors increases, rendering the ductus susceptible to vasodilation and resistant to indomethacin or ibuprofen. COX1 and COX2 expression increases with advancing maturity in the fetus (170, 171). COX inhibitors decrease vaso vasorum flow, leading to hypoperfusion and ischemic injury in the vessel wall (85). Preterm ductus also synthesizes other vasodilators such as NO, TNF-α, and IL-6 in response to wall hypoxia. Decreased ATP concentrations also prevent the ductus from contracting (172). Therefore, the preterm ductus becomes more resistant to treatment with constrictor agents (173).

### Cyclooxygenase (COX) Inhibitors

Indomethacin and Ibuprofen non-selectively inhibit cyclooxygenase enzymes; thus preventing arachidonic acid conversion to prostaglandins (Figure 2). Both drugs are associated with increased amiloride-sensitive alveolar epithelial sodium channels, suggesting improved lung water clearance and lung compliance (174). Ductal sensitivity to indomethacin increases with gestational age. Response rate to indomethacin is about 5–10% in infants <27 weeks of gestation, whereas it is almost 100% above 34 weeks (175). Half-life of indomethacin is 4–5 h longer (i.e., 17 h) in preterm infants <32 weeks of gestation. It can be given by intravenous, oral, rectal or intraarterial route. Closure rates vary from 48 to 98.5%, depending upon the dose, method and duration of administration (176, 177). Early use of indomethacin within the first few days of life decreases the risk of pulmonary hemorrhage, intraventricular hemorrhage and the need for surgical ligation, although does not affect the development of BPD (178). If needed, a second course may be given, with a success rate of 40–50% (179). Since more courses are associated with periventricular leukomalacia, repeat doses are not recommended (180). Efficacy of indomethacin declines with decreasing gestational age and becomes doubtful at 22–23 weeks of gestation (176). Indomethacin have several side effects including renal failure, oliguria, proteinuria, hyperkalemia, cerebral white matter damage, necrotizing enterocolitis, intestinal perforation and platelet dysfunction (181).

Ibuprofen is a non-selective COX inhibitor with less side effects than those of indomethacin. It can be given orally or intravenously. Elimination is slower after oral administration and the rate of closure is also higher (182). Its efficacy for ductal closure is the same as indomethacin. Major side effects include gastrointestinal hemorrhage, oliguria, high bilirubin levels, and pulmonary hypertension (183, 184). These effects may vary depending on the relevant genetic polymorphisms (185).

### Peroxidase (POX) Inhibitors

Paracetamol **(**Acetaminophen) inhibits POX enzyme which catalyzes the conversion of PG_2_ to PGH_2_, without any peripheral vasoconstriction (Figure 2). Although it is commonly discussed under the family of non-steroidal anti-inflammatory drugs, it does not have any anti-inflammatory effect (186). Its gastrointestinal tolerance is better than other NSAIDs and it lacks antiplatelet activity. Paracetamol induced POX inhibition is competitive and independent of COX activity (187). Paracetamol induced reduction in PGH_2_ production can be counteracted by lipid peroxides and PGG_2_ itself (188). The maturational difference in lipid hydroperoxide production in preterm newborns may contribute to paracetamol-induced ductal closure (188). Since the mechanism of action is different than that of indomethacin or ibuprofen, the efficacy of paracetamol in extremely low birth weight infants may be less than other NSAIDs, although solid evidence is lacking. On the other hand, its pharmacokinetics has not been well-studied in ductal closure. Inhibition of the POX dilators may lead to an increase in the pulmonary vascular resistance via peroxynitrite production (189). Furthermore, there are some data that paracetamol may synergistically enhance the inhibitory effects of other anti-inflammatory drugs on platelets (190). In preterm infants, very few cases of toxicity have been reported, which may be attributed to the low activity of cytochrome P450 in the immediate postnatal period, minimizing the formation of toxic metabolites (191). Meta-analysis comparing oral paracetamol vs. oral ibuprofen concluded that paracetamol confers comparable efficacy with fewer adverse effects (192). Paracetamol may be used as a rescue treatment for infants with persistent PDA (193). Due to the small number of studies, it is difficult do decide whether paracetamol is superior or inferior to other NSAIDs. Although there are no randomized controlled trials, there are some observational studies which suggest that paracetamol use in pregnancy or in early infancy might be associated with attention deficit/ hyperactivity disorder, autism spectrum disorder or hyperactivity symptoms. Although the exact mechanism is unknown and a cause-effect relation is difficult to establish, a small but significant odds ratio is observed in many studies (194–196). However, another study suggests that there is not an increased risk of autism in these infants at 2-year follow-up (197). There is no data on the possible long-term neurodevelopmental effects of paracetamol use in preterm infants. Therefore, paracetamol should be used cautiously until further safety data exists.

Some authors have suggested to use a combination therapy of ibuprofen and paracetamol as a first line therapy for ductal closure. However, preliminary reports suggest that the effectiveness of combination therapy does not differ compared to monotherapy of either drug (198). In another study, in infants who failed two previous cycles of monotherapy, combination therapy with oral ibuprofen and oral paracetamol was effective in 9 infants out of 12 (199). Results of an ongoing randomized controlled trial are pending (https://clinicaltrials.gov/ct2/show/NCT03103022).

Other non-steroidal anti-inflammatory drugs such as aspirin, metamizol, nimesulid, and diclophenac have constrictor effects on the ductus (200–203).

Glucocorticoids have also vasoconstrictive effects on the ductus, as well as synergistic effects with non-steroidal anti-infective drugs (NSAIDs), as a result of decreasing the sensitivity of the ductal SMCs to prostaglandins (204) (Figure 2). In the preterm fetus, elevated levels of corticosteroids is associated with decreased sensitivity of the ductus to the vasodilating effects of prostaglandins and prenatal administration of corticosteroids, mainly for the prevention of respiratory distress syndrome have also decreased the incidence of PDA in these infants (205). Similarly, postnatal corticosteroid administration may constrict the ductus (206). Angiotensin II does not have a role in ductal closure after birth (207).

There are many substances and foods with anti-inflammatory and anti-oxidant properties, such as those rich in polyphenols or flavonoids. For example, 30–40% of solid extract of green tea composes of polyphenols, mainly catechins. They exert their anti-inflammatory effects through the inhibition of endogenous prostaglandins. Similar effects have been reported with black tea, resveratrol, mate tea, orange juice and dark chocolate (207). Excessive consumption of such foods by the pregnant woman may influence the dynamics of the DA, by inhibiting COX and PG synthesis pathway. The amount of flavonoids necessary to trigger clinically significant ductal closure remains to be determined (208).

Genetic therapies may include endothelin-signaling genes or blockage of potassium channels. Channel isoforms have distinct pharmacological and physiological characteristics, making them ideal targets for ductal-selective therapies (209). Glibenclamide is a non-specific K_ATP_ channel inhibitor, used for treating diabetes. It is shown that DA constricts in response to glibenclamide, and constriction is inhibited by diazoxide, a K_ATP_ channel activator (210). Children with Cantu syndrome, which is caused by mutations in the subgroup of K_ATP_ channel genes, have PDA, often resistant to medical treatment, requiring surgical closure most of the time (16). Agonist or antagonist molecules targeting K_ATP_ channels may modulate DA tone.

Novel treatment strategies for PDA include combined use of ibuprofen and paracetamol (211), EP_4_ inhibition (212), NOS inhibition (213), enhancing ion channel expression (50), glutamate supplementation (59) especially in situations where COX inhibitors have proven to be ineffective.

### Prostaglandin E1

PGE_1_ is the main drug used for vasodilatatory effects of the ductus. PGE_1_ binds to the EP_4_receptor which increases intracellular cAMP. Milrinon is a phosphodiesterase 3 inhibitor, which can also increase cAMP levels and dilate the ductus (12).

Non-selective endothelin receptor antagonist TAK-044 and inhibition of Notch pathway may have vasodilating effects on the ductus (51, 91). Natriuretic peptide (NP) is an activator of PKG-cGMP and can prevent ductal closure also (51).

Antenatal corticosteroids are used for the maturation of preterm lung, but their effects on the development of patent ductus arteriosus, intracranial hemorrhage, and necrotizing enterocolitis are also well-studied (214). However, some reports conclude that the incidence of PDA is not effected significantly by antenatal corticosteroids (215). Antenatal steroids increase 15-PGDH activity, thereby promoting PGE_2_ breakdown (216).

Antenatal MgSO_4_ which is a calcium channel blocker may result in delayed ductal closure in infants (217). However, recent evidence in extremely low birth weight infants suggests that antenatal MgSO_4_ exposure is not associated with an increased risk of hemodynamically significant PDA; in fact, it may be associated with a decreased likelihood of hemodynamically significant PDA (218). Diazoxide, which is a K_ATP_ channel activator may induce ductal opening (173). Furosemide increases renal PGE_2_ production, thus dilating the ductus in animals but its effect is limited in humans (219).

## Conclusion

Ductus arteriosus is an intriguing and complex vessel with many distinct features. Understanding the precise mechanisms of regulation of the ductus is essential in order to improve individual pharmacological treatments. The debate about PDA should not be “treat all” or “treat none,” but rather, “who to treat.” This approach necessitates individualized treatment. Treatments targeting endogenous ductus regulation pathways other than prostaglandins, such as cell cycle regulators, transcription factors, and epigenomic regulators should be developed. Since vascular constriction and ductal wall remodeling are required for complete closure, treatments that favor vasoconstriction and remodeling would also be ideal for infants with persistent PDA. Novel therapies for ductal closure in preterm infants should also focus on a clear understanding of genes which are operative on ductal patency, as new genome-wide studies uncover genes associated with PDA. Evolving pediatric clinical pharmacology heralds next generation solutions for unresolved issues of today, such as drugs with selective DA effects, without any side effects.

## Author Contributions

FO is responsible for the design, research, writing, and editing of the manuscript.

## Conflict of Interest

The author declares that the research was conducted in the absence of any commercial or financial relationships that could be construed as a potential conflict of interest.
